# Intelligent Nanomaterials for Solar Energy Harvesting: From Polar Bear Hairs to Unsmooth Nanofiber Fabrication

**DOI:** 10.3389/fbioe.2022.926253

**Published:** 2022-07-25

**Authors:** Qingli Wang, Ji-Huan He, Zhi Liu

**Affiliations:** ^1^ Department of Postgraduates, Shanghai University of Engineering Science, Shanghai, China; ^2^ School of Mathematics and Information Science, Henan Polytechnic University, Jiaozuo, China; ^3^ National Engineering Laboratory for Modern Silk, College of Textile and Clothing Engineering, Soochow University, Suzhou, China; ^4^ School of Textile and Garment, Anhui Polytechnic University, Wuhu, China

**Keywords:** biomimetic, polar bear hair, energy absorption, selective light absorption, moth-eye effect, bubble electrospinning

## Abstract

Polar bears can live in an extremely cold environment due to their hairs which possess some remarkable properties. The hollow structure of the hair enables the bear to absorb energy from water, and the white and transparent hairs possess amazing optical properties. However, the surface morphology function of bear hairs has been little-studied. Herein, we demonstrate that the micro-structured scales distributed periodically along the hair can absorb maximal radiative flux from the Sun. This polar bear hair effect has the ability for the hair surface not to reflect radiation with a wavelength of about 500 nm. Mimicking the polar bears’ solar performance in the fabrication of nanofibers will certainly stimulate intelligent nanomaterials for efficient solar energy absorption. Therefore, a new technology is discussed in this work for the fabrication of periodic unsmooth nanofibers toward solar energy harvesting.

## Introduction

Recently, various devices have been developed for energy harvesting, such as the nanofluids (He and Elazem, 2022), the spring-pendulum systems (Wu et al., 2018; He et al., 2022a), and the low-frequency vibration systems (Zhang and Cai., 2012; He C.-H. et al., 2021; He et al., 2022b). In addition to the abovementioned methods, the nanotechnology for solar energy harvesting (Satharasinghe et al., 2020) is totally new and is quite promising. Though solar energy harvesting has attracted much attention due to the inexhaustible green energy, its efficiency is relatively low. Interestingly, some natural animals have a special ability to absorb solar energy with extremely high efficiency, benefiting from the amazing surface morphology of their hairs, for example, the wolverine (gulo-gulo) hair (Liu et al., 2018).

The polar bear (*Ursus maritimus*) is the largest predator in the Arctic region. As a kind of marine mammal, the animal is born on land but spends most of its time in the sea to absorb energy from water through its hairs (He et al., 2011; Jia et al., 2017). In order to survive in a harsh environment as low as −50°C in the Arctic, this huge animal has an extensive fat layer of up to 10 cm and bulky furs, which help protect against the cold surrounding. Polar bear fur consists of a layer of dense underfur and an outer layer of guard hairs, which are transparent and white in color (Bechshøft et al., 2012; Dietz et al., 2013). The white hairs contribute to camouflaging the bear in the snow and ice-covered environment (Ferguson et al., 1998; Stegmaier et al., 2009). As a protein fiber, the polar bear hair is not much different in appearance from other protein fibers such as the wool fiber (Fan et al., 2019) and down fiber (Yang et al., 2011). Much attention has been paid to the optical properties (Lavigne and Øritsland, 1974bib_Lavigne_and_Øritsland_1974; Grojean et al., 1980 and 1981; Koon, 1998) and chemical properties of polar bear hairs, and many biomimetic designs were proposed, including thermally insulating fabrics (Cui et al., 2018), textile solar light collectors (Bahners et al., 2008), and polar bear hair–based solar sensors (Tributsch et al., 1990). Many researchers have studied hair cortisol concentration (Mislan et al., 2016), which is considered a biomarker. Furthermore, the morphology and structure, especially the hollow structure of polar bear hairs, have also been studied extensively (Zhang et al., 2009). The fractal theory is a useful tool to reveal its biomechanism (Wang et al., 2015; Wang et al., 2018). However, energy absorption with regards to the scale distribution on polar bear hairs has not been studied yet, and this study intends to state its energy absorption based on the hair’s morphology. Many research studies have revealed that the graphene distribution in a composite affected its properties greatly (Zuo and Liu, 2021; Zuo, 2021). Geometry is always the main factor affecting materials’ properties (He, et al., 2021b) so that the energy absorption property of the polar bear hair.

## Materials and Methods

Nano-scale surface morphology greatly affects a surface’s chemistry property (Marmur, 2004; Li X.-X. et al., 2021), friction property (Cao et al., 2021; Bains et al., 2020), and reflection property (Selkowitz 2021). According to the geometric potential theory (Peng and He, 2020; Han and He, 2021), a nano-scale surface can produce high geometric potential. It was reported that Fangzhu’s nano-scale surface can collect water molecules from the air (He and El-Dib, 2021; Wu et al., 2021). Gecko adhesion and the mimicking smart adhesion can also be explained by its nano-scale spatulas (Wang et al., 2019; Li et al., 2020). Here, an experiment is designed to study the nano-scale surface morphology of polar bear hairs.

### Experimental Materials

The hair was obtained from a 2-year-old male polar bear in the Laohutan Pole Aquarium in Dalian, China. Polyvinyl alcohol (PVA, 1750 ± 50) was purchased from Sinopharm Chemical Reagent Co., Ltd. (Shanghai, China).

### Experimental Instruments

The JEOL JSM-5600LV scanning electron microscope (SEM) with a magnification of 18-300,000 (Japan Electronics Co. Ltd.) and the S-4800 field emission scanning electron microscope (FE-SEM) (Hitachi Ltd., Japan) with resolutions of 1.0 nm (15 kV), 2.0 nm (1 kV), and 1.4 nm (1 kV, Deceleration mode) were used in our experiment. The scale density (scale/mm) and scale thickness on the hair surface were measured through the SEM images.

### Experimental Process

First, we rinsed the samples with distilled water to remove debris from their surface. Second, the samples were washed with 0.1 M phosphate buffer three times (15 min per time). Third, the samples were fixed on the sample stage and sprayed in the ion sputter instrument. Finally, the samples were evaluated under the SEM and FE-SEM. In the electrospinning process, the PVA was dissolved in an aqueous solution (8 wt%) at 98°C for 4 h. The spinning parameters were as follows: the voltage was 20 kV, and the collector distance was 25 cm. After the spinning process, the resulting membrane was stretched with a draw ratio of 1.5 times. The draw process was carried out using a universal testing machine (Instron 3,365, Instron, Norwood, MA) (gauge length: 20 mm and cross-head speed: 0.2 mm/s) at 25 ± 0.5°C and 60 ± 5% relative humidity.

### Nanoscale Surface Morphology

Before studying the polar bear hair effect, we give a brief introduction to the moth-eye, which can absorb all night light (Wilson and Hutley, 1982). This property is important for the nocturnal insect to escape from predators.

**FIGURE 1:**
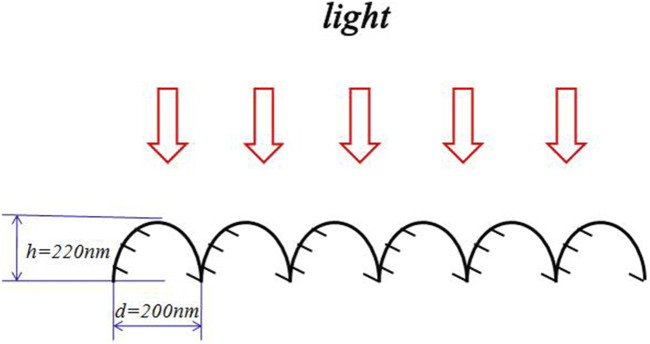
Schematic diagram of the moth-eye effect (Nosonovsky and Bhushan,^2013^).

Figure 1 is the schematic diagram of the moth-eye effect. The height of protuberances is about *h* = 220 nm, and the diameter of the microtrichia is about *d* = 200 nm.

It was reported that the reflectance is very low for wavelengths 2d<λ<2.5h (Nosonovsky and Bhushan, 2013), meaning that the wavelengths between 440 and 550 nm are all absorbed by the nocturnal insect.

The surface geometry of the moth eye is periodic, and it was used for the biomimetic design of an optically transparent microwave absorber with a periodic array of properly shaped glass caps (Kwon et al., 2021). A similar phenomenon occurs in the polar bear hair, and we carried out an experiment to study the morphology of the hair surface.

## Results and Discussion

**FIGURE 2:**
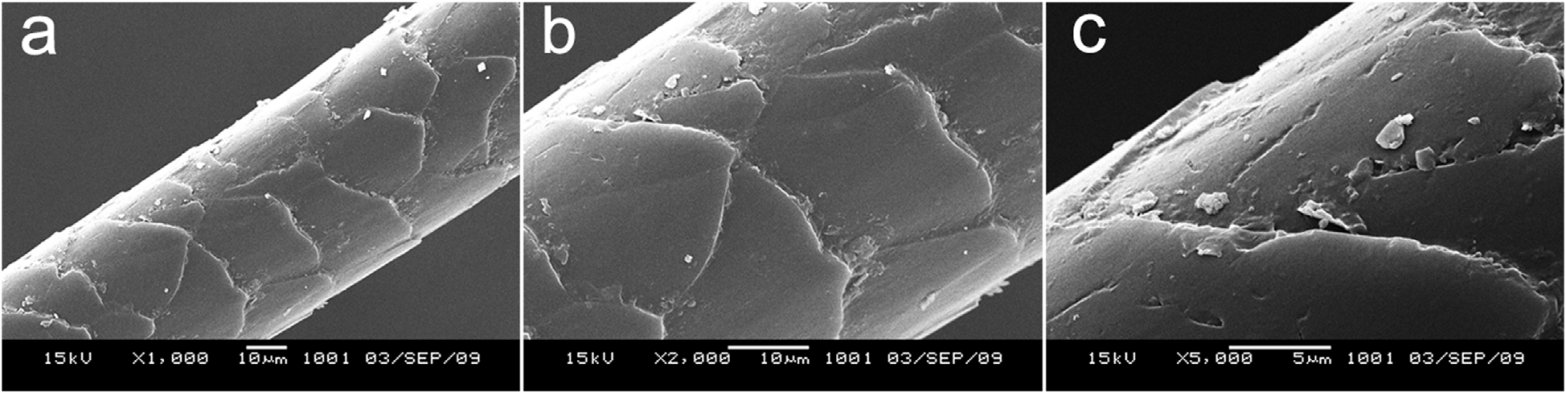
Polar bear hair surface structure by SEM with different magnifications **(A)** ×1,000, **(B)** ×2,000, and **(C)** ×5,000.

**FIGURE 3:**
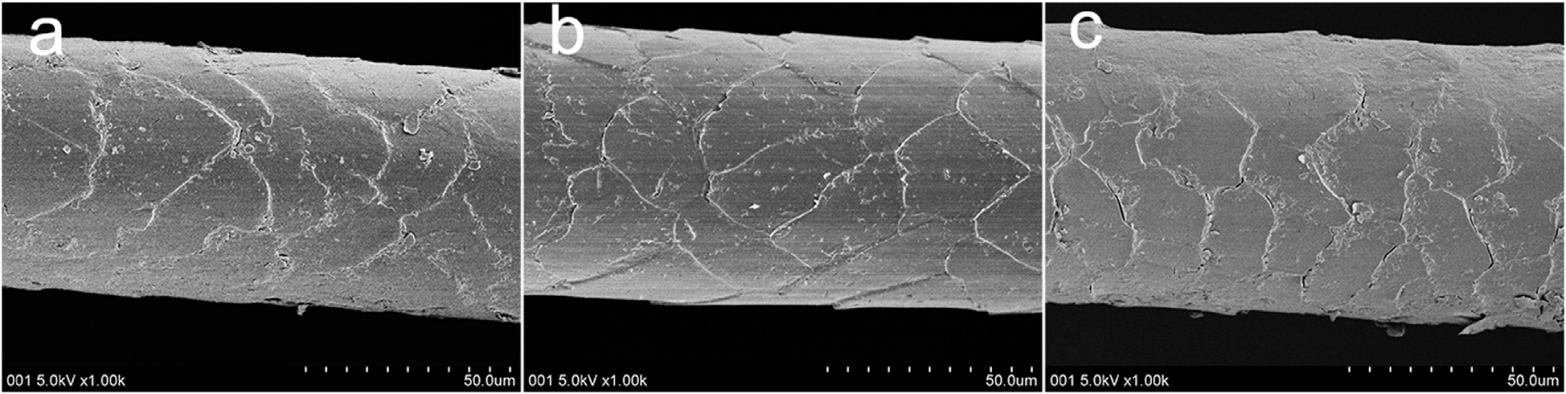
Polar bear hair surface structure by FE-SEM **(A-C)** different locations on the hair surface.

Figure 2 shows the morphology of the polar bear hair surface structure. Figures 2A–C are SEM images of the same hair at the magnifications × 1,000, × 2,000 and × 5,000, respectively. Figure 3 shows the FE-SEM of the polar bear hair surface structure. As we can see from Figures 2 and 3, the surface of polar bear hair fibers is not smooth, and there is a scale structure similar to the surface of wool fibers. Meanwhile, these figures also show that the polar bear hair fibers were covered with periodic scales in regular shapes. Scale density was relatively small, 70–90 scales/mm, and the scale edges seemed to be wavy or serrated. The scales are thicker at the top, and the scale thickness is about 0.5 µm.

**FIGURE 4:**
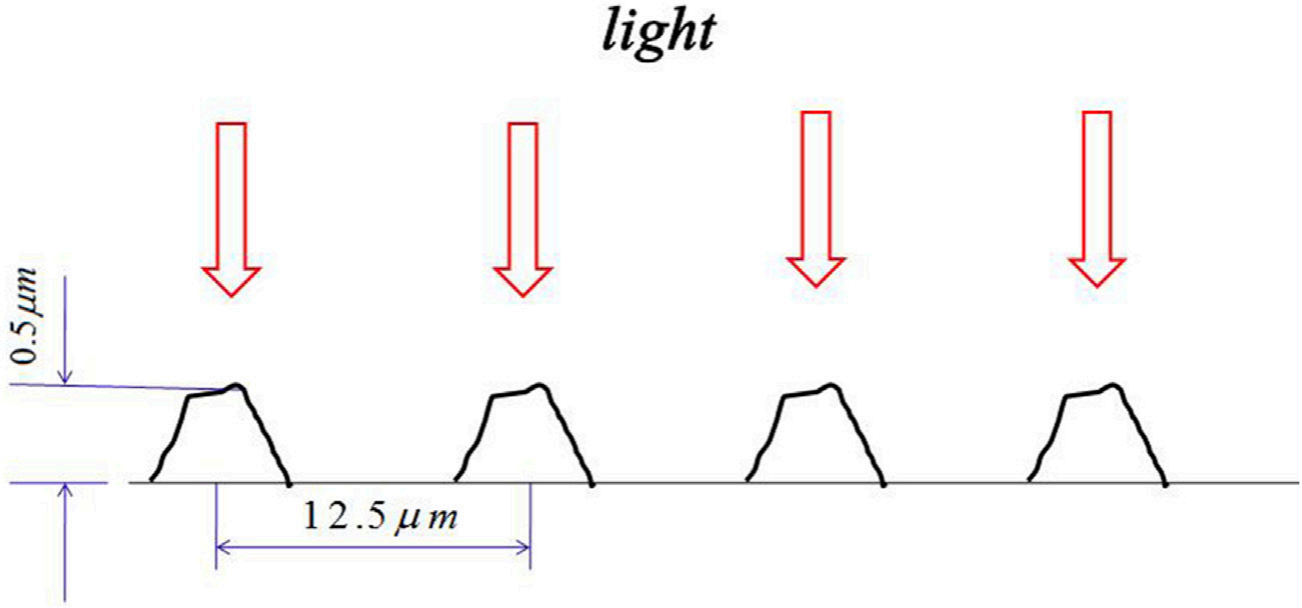
Schematic diagram of the polar bear hair effect.

Similar to the moth effect (Nosonovsky and Bhushan, 2013), polar bear hair enables the animal to absorb as much energy as possible from natural light. According to Figures 2 and 3, polar bear hair can be geometrically illustrated, as shown in Figure 4.

**FIGURE 5:**
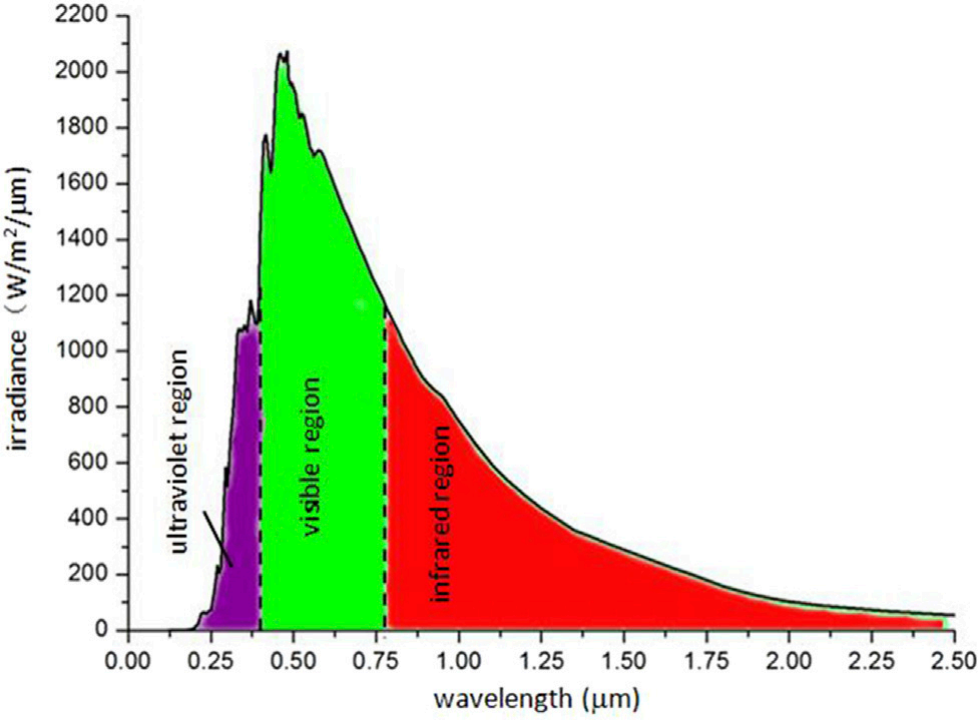
Maximal solar spectral irradiance at 500 nm wavelength.

The periodicity of the surface morphology of polar bear hair is similar to that of the moth eye and also shows a similar optical property to absorb light energy. Polar bear hairs are white and transparent to convert light energy to its body. The hair surface morphology (Figure 4) can increase transmission and reduce reflection. If the thickness of the scales is almost equal to the light wavelength (Nosonovsky and Bhushan, 2013), the light will not be reflected. Our experimental data reveal that the scale thickness is about 500 nm, corresponding to the spectrum of 500 nm wavelength. According to the laws of radiative heat transfer, the radiative flux from the Sun maximizes at a wavelength of about 0.5 μm (Scamarcio et al., 1997; Thuillier et al., 2003) (Figure 5). The polar bear hair effect is the ability of a micro-structured optical surface not to reflect light with the highest energy.

**FIGURE 6:**
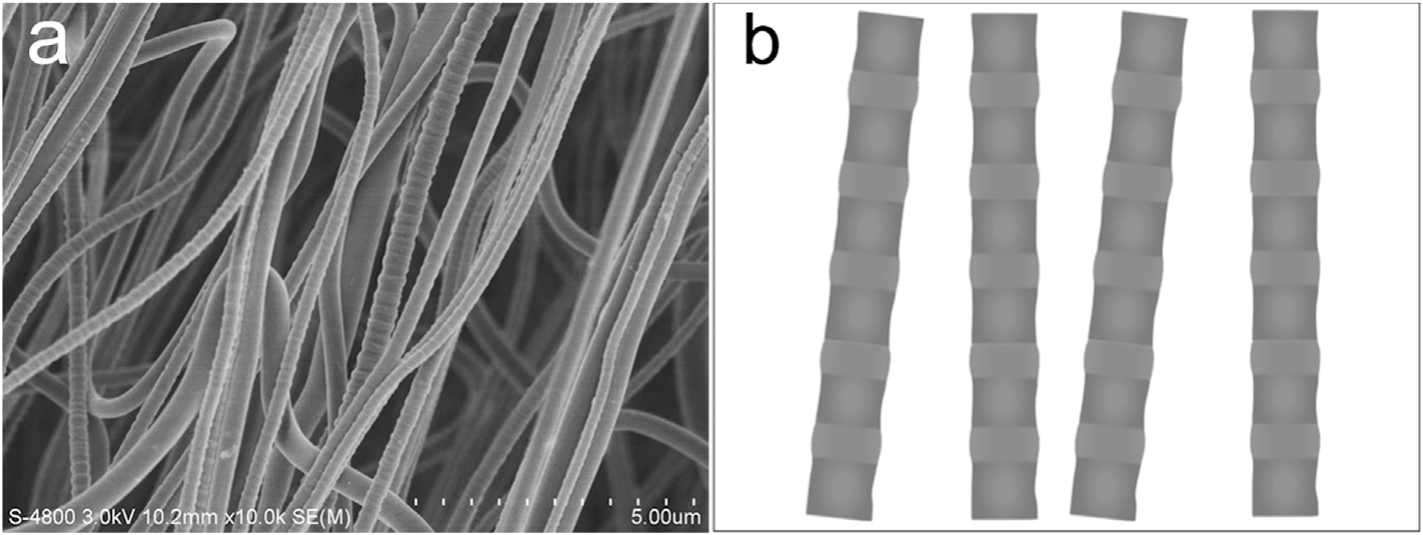
**(A)** Morphology of the periodic unsmooth PVA nanofiber. **(B)** Schematic diagram of the periodic bamboo-like unsmooth PVA nanofiber.

The general approaches to fabricating smooth nanofibers are electrospinning (Gao et al., 2021; Liu et al., 2021) and bubble electrospinning (He and Qian, 2022; Qian and He, 2022). However, Lin, et al., 2021 suggested a general strategy for the fabrication of unsmooth nanofibers. Yao and He (2020) used the geometric potential theory to control the surface morphology of nanofibers. These references suggested that the unsmooth nanofibers can be fabricated by the electrospinning method. Here, inspired by the natural polar bear, a new technology is discussed for the fabrication of periodic unsmooth nanofibers for solar energy harvesting (Liu et al., 2015). As shown in Figure 6, the resulting PVA nanofibers were endowed with peculiar morphology with a periodic unsmooth surface after being stretched (Figure 6A). The bulges with a diameter of about 80 nm were successfully constructed and periodically spread along the nanofiber axis (Figure 6A), exhibiting a similar appearance to natural bamboo (Figure 6B). The mutational surface morphology from a smooth surface to a bamboo-like unsmooth surface by the stretching method suggests a promising strategy to mimic the polar bear’s solar performance in the fabrication of intelligent nanomaterials for efficient solar energy–absorption.

The nanofibers with a periodic unsmooth surface are potentially of great technological interest for the development of solar energy absorption, and their other applications include invisibility devices, electronic sensors, applied surface science, photonics, physics, microelectronics, nanomaterials, advanced textile, photothermo-promoted nanocatalysis, photothermal semiconduction, photoactivatable cancer immunotherapy, and environmental science (Li et al., 2019; Li X. et al., 2021; Li J. et al., 2021; Yang et al., 2021). We anticipate that this article will be a starting point for more sophisticated studies of intelligent nanomaterials for solar energy harvesting for solar cells (Pavlovic et al., 2021; Alshikhi and Kayfeci 2022) or solar collector systems (Al-Rabeeah et al., 2022). The periodic structure of nanomaterials *via* bioinspiration for energy gives many promises and great challenges (Gong et al., 2019).

## Conclusion

Similar to the moth-eye effect, polar bear hair characteristics along the longitudinal direction were studied by means of SEM and FE-SEM, respectively. The result shows that the micro-structured scales distributed periodically along the hair can absorb maximal radiative flux from the Sun. Mimicking the polar bear’s solar energy harvesting property, we fabricated nanofibers with a periodic unsmooth surface, which exhibits the potential of stimulating intelligent nanomaterials for efficient solar energy absorption. The bio-mimic design of solar energy materials by bubble electrospinning can be used in the infrared stealth technology. Polar bear hair–inspired biomaterials with selective light absorption will attract much attention in the future.

## Data Availability

The original contributions presented in the study are included in the article/Supplementary Material; further inquiries can be directed to the corresponding authors.
